# Carcinome vésical compliqué de métastase irienne

**DOI:** 10.11604/pamj.2014.19.232.4245

**Published:** 2014-10-31

**Authors:** Rajae Derrar, Nourredine Boutimzine

**Affiliations:** 1 Université Mohammed V Souissi, Service d'Ophtalmologie, Hôpital des spécialités CHU Rabat, Maroc

**Keywords:** Carcinome vésical, métastase irienne, œdème palpébral, bladder carcinoma, iris metastasis, eyelid edema

## Image en medicine

Patient âgé de 44 ans, Opéré il y'a 1 an et demie pour tumeur de la vessie: Carcinome urothélial infiltrant grade 3, suivi de 10 séances de radiothérapie vu aux urgences é ans et demi plus tard pour baisse d'acuité visuelle progressive au niveau de l’œil gauche avec douleur et rougeur. L'examen de l’œil droit est normal, au niveau de l’œil gauche une acuité visuelle limitée à Compte les doigts de près avec œdème palpébral, hyperhémie conjonctivale et larmoiement important, un Tonus oculaire normal. L'examen à la lampe à fente trouve de fins précipités rétrocornéens Jaunâtres, une formation tumorale à point de départ irien, blanchâtre, d'environ 4mm/4mm, bosselée, vascularisée cachant la majeure partie de l'aire pupillaire, entourée d'un magma fibreux (flèche rouge) avec un petit nodule irien en temporal vers 2h (flèche blanche) de même aspect macroscopique, des Synéchies iridocapsulaires. Une échographie faite montre une masse irienne de 3 mm/4 mm Vitré clair, une choroïde d’épaisseur normale et la rétine en place. Une cytoponction de la tumeur après paracentèse: montre la présence de multiples cellules carcinomateuses avec structure papillaire évoquant l'origine métastatique. On demande un bilan radiologique d'extension comprenant une radio pulmonaire, une échographie abdominale et une scintigraphie osseuse qui montre de multiples foyers osseux Vertébraux et iliaques métastatiques. Le patient a été adressé en oncologie pour chimiothérapie. Les métastase iriennes secondaire à un carcinome vésical sont rares, le traitement étant souvent palliatif, le pronostic est mauvais et l'espérance de vie réduite.

**Figure 1:**
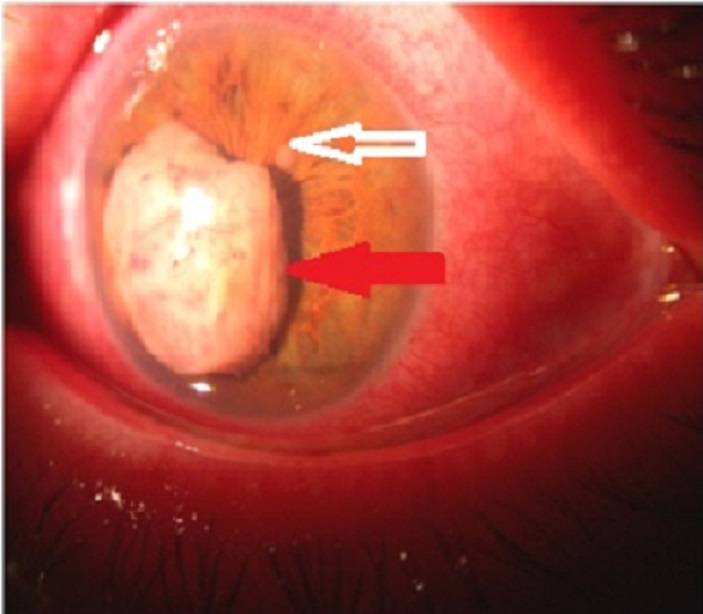
Métastase irienne en chambre antérieure (fléche blanche: nodule irienne; fléche rouge: métastase irienne)

